# Synthesis and Characterization of Sucrose and Ammonium Dihydrogen Phosphate (SADP) Adhesive for Plywood

**DOI:** 10.3390/polym11121909

**Published:** 2019-11-20

**Authors:** Zhongyuan Zhao, Shijing Sun, Di Wu, Min Zhang, Caoxing Huang, Kenji Umemura, Qiang Yong

**Affiliations:** 1 College of Furnishings and Industrial Design, Nanjing Forestry University, Nanjing 210037, China; nfuwudi@163.com; 2 College of Material Science and Engineering, Nanjing Forestry University, Nanjing 210037, China; sunsj-611@163.com; 3 Laboratory of Sustainable Materials, Research Institute for Sustainable Humanosphere, Kyoto University, Gokasho, Uji, Kyoto 611-0011, Japan; zhang888@rish.kyoto-u.ac.jp; 4 College of Chemical Engineering, Nanjing Forestry University, Nanjing 210037, China; szxapy@163.com

**Keywords:** eco-friendly adhesive, sucrose, ammonium dihydrogen phosphate, plywood

## Abstract

The development of eco-friendly adhesives for wood composite products has been a major topic in the field of wood science and product engineering. Although the research on tannin-based and soybean protein-based adhesives has already reached, or at least nears, industrial implementation, we also face a variety of remaining challenges with regards to the push for sustainable adhesives. First, petroleum-derived substances remain a pre-requisite for utilization of said adhesive systems, and also the viscosity of these novel adhesives continues to limit its ability to serve as a drop-in substitute. Within this study, we focus upon the development of an eco-friendly plywood adhesive that does not require any addition of petroleum derived reagents, and the resultant liquid adhesive has both high solid contents as well as a manageably low viscosity at processing temperatures. Specifically, a system based on sucrose and ammonium dihydrogen phosphate (ADP) was synthesized into an adhesive with ~80% solid content and with viscosities ranging from 480–1270 mPa·s. The bonding performance of all adhesive-bound veneer specimens satisfied GB/T 9846-2015 standard at 170 °C hot pressing temperature. To better explain the system’s efficiency, in-depth chemical analysis was performed in an effort to understand the chemical makeup of the cured adhesives as well as the components over the time course of curing. Several new structures involving the fixation of nitrogen speak to a novel adhesive molecular network. This research provides a possibility of synthesizing an eco-friendly wood adhesive with a high solid content and a low viscosity by renewable materials, and this novel adhesive system has the potential to be widely utilized in the wood industry.

## 1. Introduction

Nowadays, the development of bio-based materials has been a main topic in the field of material science and technology [1,2,3,4]. Compared to novel bio-based materials, the traditional wood-based materials are more heavily utilized by societies worldwide. Most of these materials (such as particleboard [5], plywood [6,7,8,9], medium density fiberboard [10], and oriented strand board [11]) are manufactured by bonding wooden elements with adhesives derived from petroleum. Examples of such adhesives include urea-formaldehyde, phenolic-formaldehyde, and melamine-formaldehyde resins [12,13,14]. However, with use of these resins comes concerns regarding volatile organic compound (VOC) emissions, potential health hazards, and non-biodegradability [15,16]. To avoid the utilization of formaldehyde, although the isocyanate-based resins are developed and used in wood industry, the raw materials of these adhesives are derived from fossil resources, which are not renewable and continue to decrease. These concerns have spurred manufacturers of wood based materials towards critical research to establish product portfolios that contain more sustainable and green products within. Specifically, formaldehyde-free and renewables-based wood adhesives are a major subject of ongoing investigations [17,18].

**Figure 1:**
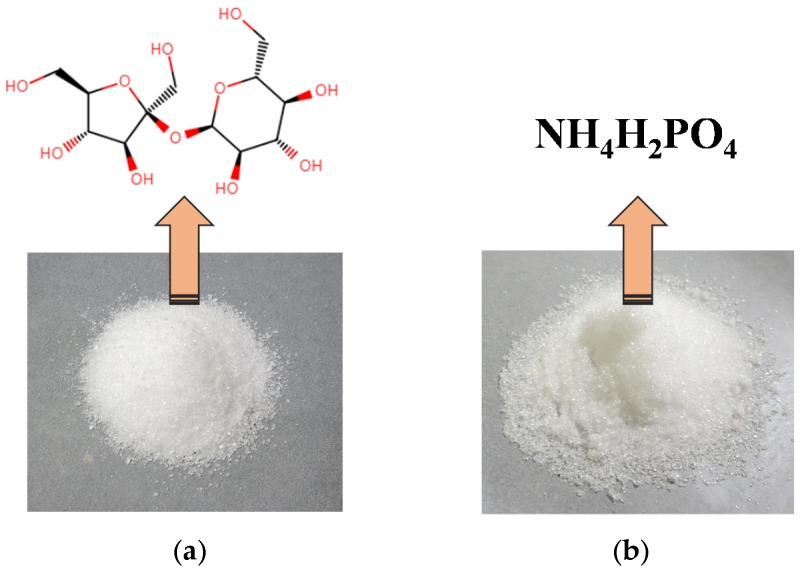
Appearance and chemical formula of (**a**) sucrose and (**b**) ammonium dihydrogen phosphate.

Sucrose (Figure 1a) is one of the world′s most abundantly produced organic compounds, and it is available at a very high level of purity while still remaining at low cost. The most common industrial consumers are in the beverage and food industries, but it can also finds some applications in the chemical processing industries [19,20]. Sucrose is considered to be a complex multifunctional molecule that is highly oxygenated. It contains eight reactive hydroxyl groups and two anomeric carbon atoms, properties that allow for it to be malleable when generating valuable derivatives from it. In fact, most attempted transformations are prone to making complex micromolecule mixtures that are highly reactive (such as 5-HMF and HMF) [21]. In addition, sucrose is a reasonably soluble compound, and in only a limited number of solvents and along with the aid of a catalytic can this transform reaction occur. This solubility provides the capability of being able to synthesize a high solid content solution based on a cross-linking prone polymer derived entirely from sucrose [20]. 

Recently, some researches presented novel adhesives based on organic polymers (tannin and lignin) and phosphate, which could form cross-linkage during the heating process [22,23]. In our recent research, a similar adhesion system was also developed. A mixture of sucrose and ammonium dihydrogen phosphate (ADP, Figure 1b) was dissolved in distilled water at room temperature and successfully applied to a particleboard product. The novel board products bonded by sucrose-ADP adhesive demonstrated the physical properties and water resistance that satisfied JIS standards [24], results that suggest that this sort of adhesive system could be suitable for introduction into a variety of wood composite products. According to some research on the food chemistry, ammonium salts could react with reducing sugars to form colorants in the food and beverage industry, and the products of this reaction are polymeric materials [25,26]. Based on the positive results of our previous research, herein we have further investigated this bio-based adhesive system to create a product that can be synthesized with minimal limited water that has high enough solids content and viscosities tolerable for introduction into existing wood product manufacturing operations. The goal of this work is to push forward an intriguing new technology that could alleviate the range of concerns that exist for wood product consumers based on the industry’s consistent utilization of problematic petroleum-based adhesive systems.

## 2. Materials and Methods 

### 2.1. Materials

Sucrose (analytical-grade reagent) and ammonium dihydrogen phosphate (analytical-grade reagent) were purchased from Sinopharm Chemical Reagent Co., Ltd (Shanghai, China). Each reagent was vacuum-dried at 60 °C until constant mass prior to usage in experiments. Poplar veneers (species: *Populus tomentosa* Carr) were purchased from Zuogezhuang, Hebei Province of China, and the product method was rotary cutting. 

### 2.2. Synthetic Procedures of SADP Adhesives

**Figure 2:**
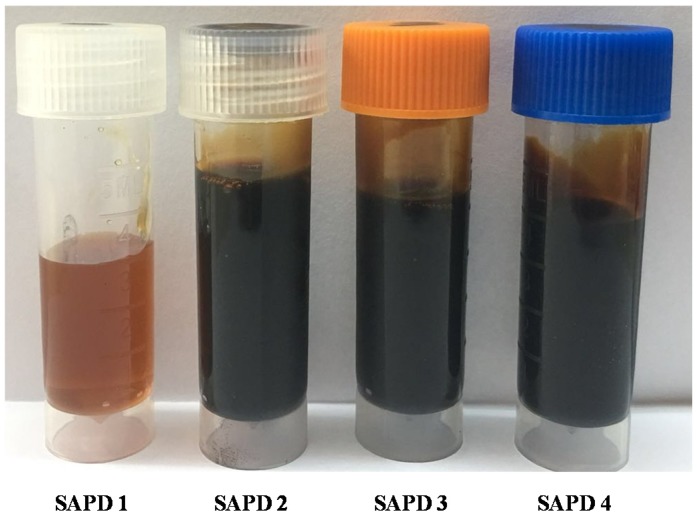
Appearance of synthesized SADP adhesives.

Sucrose and ADP were first mixed with an 85/15 proportion, and then were translated to 5.7 parts sucrose per 1 part ADP. These reagents were next poured into a three mouthed bottle with distilled water. The weight with distilled water included was calculated based on the water solubility of sucrose at 90 °C [27] (Equation (1)). This mixture was then heated in an oil bath at 90 °C for certain time while it was sheared at 180 rpm/min to facilitate synthesis of the sucrose-ADP (SADP) adhesive. The pH of the adhesives was measured at 30 °C using a Leici pH meter PHBJ-206 (Leici, Shanghai, China). The viscosity of the adhesives was measured using a HAAKE rotational rheometer MA S60 (HAAKE CO., Karlsruhe, Germany). Specifically, 2 mL of each adhesive were loaded onto a flat plate set at 30 °C. The measuring geometry (C60 2°/Ti-02170027) was next lowered, and the testing mode was CR using 100/s shear rate. Each viscosity test experiment lasted 300 s until the viscosity values stabilized. A total of 80 viscosity measurements were acquired over the duration of analysis, while the final viscosity was defined as the average values of last 40 data points. The results of pH and viscosity are shown in Table 1. The appearance of synthesized SADP adhesives is shown in Figure 2. All the synthesized SADP adhesives were sealed and stored at room temperature for at least three days before further research was conducted involving them.

(1) $$Weight\;of\;distilled\;water\;\left(g\right)=\frac{Weight\;of\;sucrose\;\left(g\right)\;\times \;100\;\left(g\right)}{\;Water\;solubility\;of\;sucrose\;at\;90\;{}^{\circ}C}$$

### 2.3. Bond Performance

#### 2.3.1. Manufacture of Plywood

The synthesized SADP adhesives were utilized to manufacture a three-layer plywood (300 mm× 300 mm) in order to evaluate the bonding performance. Moisture content and thickness of the veneers were 9.8–11% and 1.5 mm, respectively. SADP adhesives were applied to the core veneer at a spread rate of 140 g/m^2^ for a single veneer surface. The coated veneer was then stacked between two uncoated veneers so that the grain direction of the two adjacent veneers was perpendicular to each other. The assembled three-layered plywood with each SADP adhesive was next hot-pressed for 7 min at 150, 170, and 190 °C. In total, 12 plywood pieces were produced, and the average thickness of the manufactured three-layer plywood was 4.6 mm. Detailed formulations of the conventional adhesives are shown in Table 2.

#### 2.3.2. Shear Strength Measurement

The prepared plywood was cut into standard tensile shear test specimens according to China National Standards (GB/T 9846.7-2004). Twelve plywood specimens (10 cm × 2.5 cm) were cut from each manufactured plywood and six specimens were submerged into water at 63 ± 2 °C for 3 h. Then the tensile shear strength of the plywood was measured at dry and wet conditions under a loading rate of 1.0 mm/min. Each plywood test was carried out in twelve replications, and the average values, standard deviations, and average wood failure were calculated. Statistical significance was considered for *p* values < 0.05.

### 2.4. Curing Behavior

#### 2.4.1. TG Analysis

A measure of 100 g of each prepared adhesive was poured into glass vials, and then freeze-dried to obtain solid uncured adhesives. These uncured adhesives were analyzed by thermogravimetric analysis (TGA) using a Netzsch STA 449 F5 Jupiter^®^ (Netzsch, Selb, Germany). The samples were scanned from room temperature to 400 °C at a rate of 10 °C/min under nitrogen.

#### 2.4.2. Viscosity-Temperature Characteristics

Viscosity-temperature curves of the SADP adhesives were measured by a HAAKE rotational rheometer MA S60 (HAAKE CO., Karlsruhe, Germany). In this test, 2 mL of each adhesive was applied to the flat plate, while the measuring geometry was C60 2°/Ti-02170027. The temperature range was set from 30 to 160 °C, and the testing mode was CR with 100/s shear rate. Each experiment sustained 900 s, providing 100 data points used to form a curve.

### 2.5. Synthesis and Curing Mechanism

#### 2.5.1. High-Performance Liquid Chromatography (HPLC) Analysis

The chemical composition of the liquid SADP adhesives were measured using Agillent 1260 high-performance liquid chromatography (HPLC) (Agillent Technologies Inc., Santa Clara, CA, USA). Before the measurement, the adhesive solutions were diluted 300 times. The HPLC system was equipped with an HPX-87H ion exclusion column (300 mm × 7.8 mm), degasser, pump, and refractive index (RI) detector. HPLC grade milli-Q water was used as the eluent at a flow rate of 0.6 mL/min and at a column temperature of 55 °C.

#### 2.5.2. Attenuated Total Reflection-Fourier Transform Infrared Spectra (ATR-FTIR)

ATR-FTIR spectra were acquired to probe for chemical changes between (i) the uncured SADP adhesives (freeze dried), and (ii) the insoluble mass of SADP 2 (obtained by curing at different heating temperatures for 7 min, by boiling in the distilled water for 4 h, and finally, by drying at 60 °C for 15 h). Infrared spectra were obtained using an ATR-FTIR spectrophotometer (Nicolet iS10, Thermo, Waltham, MA, USA), and were recorded with an average of 32 scans at a resolution of 4 cm^−1^.

#### 2.5.3. X-ray Photoelectron Spectroscopy (XPS) Analysis

X-ray photoelectron spectroscopy (XPS, Kratos AXIS Ultra DLD, Manchester, UK) was performed to evaluate the chemical element composition of the cured SADP adhesives. The survey-scan mode was applied to record the low-resolution spectra with a binding energy region of 0–1200 eV. In addition, the high-resolution spectra (C 1s and N 1s) derived from the insoluble mass of SADP 2 were recorded at the binding energy region of 277–296 and 392–408 eV, respectively.

## 3. Results and Discussion

### 3.1. Shear Strength of Plywoods Prepared Using SADP Adhesives

**Figure 3:**
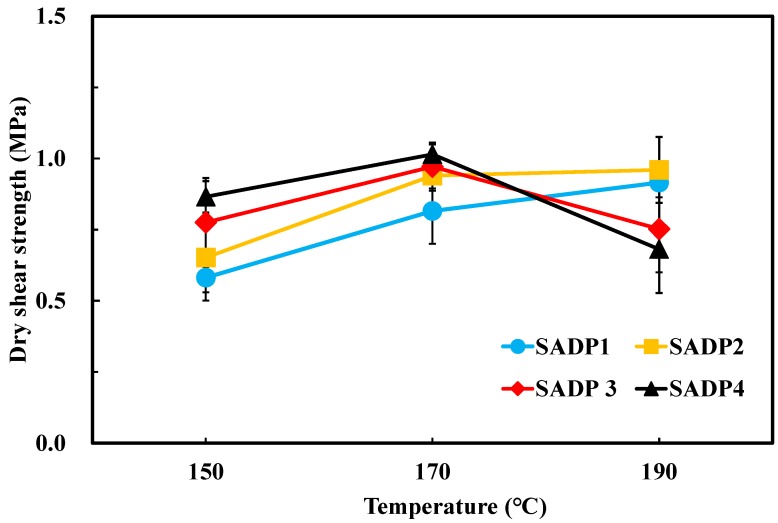
Dry shear strength of the plywood bonded with SADP adhesives.

**Figure 4:**
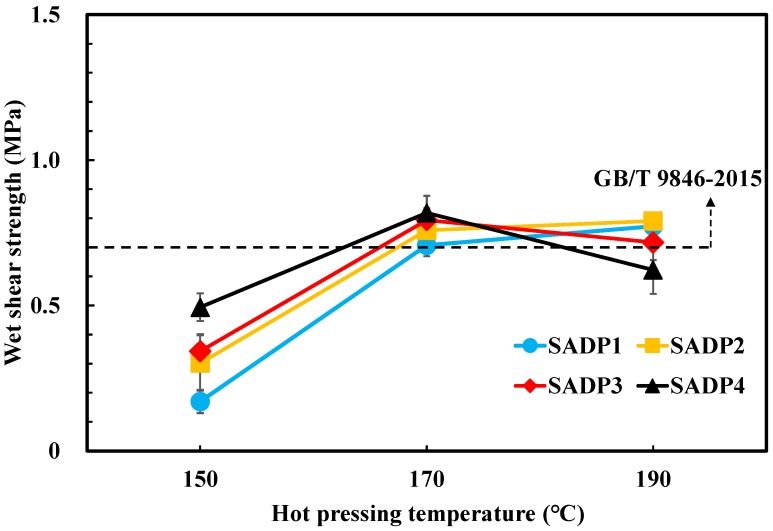
Wet shear strength of the plywood bonded with SADP adhesives.

To evaluate the bond performance of SADP adhesives, three-ply plywood was fabricated using the synthesized SADP adhesives, and the results of dry and wet shear strength are shown in Figure 3 and Figure 4. The profiles visualized in both figures show that the shear strength of the boards bonded with SADP 1 and SADP 2 exhibited a positive relationship with hot pressing temperature. However, the plywood manufactured using SADP 3 and SADP 4 shows a negative correlation trend at 190 °C, which was observed as a decrease in the mechanical properties compared with the boards bonded at 170 °C. Considering the results shown in Table 3, both the dry and wet wood failure of the boards bonded with SADP 3 and SADP 4 at 190 °C was 90 and 100%, respectively. This performance result indicates that the reason that the shear strength reduced for these two adhesives could be due to decreasing veneer strength. This could be related to the pH values of SADP 3 and SADP 4, measured as 2.35 and 2.01, respectively. This lowered pH may have caused the liberation in the liquid resin of phosphoric acid and pyrophosphoric acid produced from the ADP [28], two components that can deteriorate the wood elements at the high pressing temperatures that were investigated [29]. Regarding wet shear strength, the average values of all the samples hot pressed at 170 °C satisfied to the China National Standard (GB/T 9846-2015). It could be found that the maximum wet shear strength was obtained from SADP 4 (0.82 MPa), and variance analysis (ANOVA) revealed no significant (*p* > 0.05) difference of wet shear strength between SADP 3 and 4 hot pressed at 170 °C; however, although ANOVA analysis showed a significant difference between SADP 2 and the maximum values, judging from the holistic performance of the plywood bonded with SADP adhesives and the pH values of each adhesive, the optimal synthesis time and hot pressing temperature in this study are considered as 2 h and 170 °C, respectively.

### 3.2. Analysis of SADP Curing Behaviors

**Figure 5:**
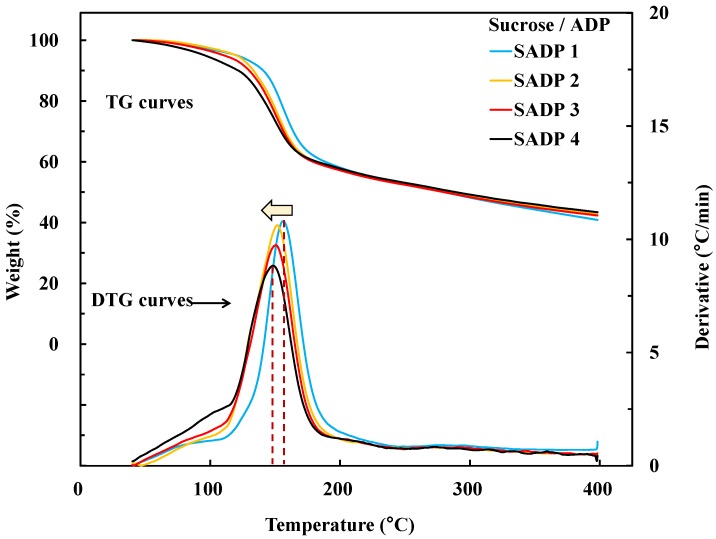
TG and DTG curves of each SADP adhesive.

TG analysis and viscosity-temperature analyses were performed to investigate the curing behavior of the SADP adhesives. Figure 5 shows the thermogravimetric (TG) and derivative TG (DTG) curves of each SADP adhesive. From the TG results, it was found that the preliminary weight loss temperature of all the SAPD adhesives was approximately 100 °C. However, the preliminary weight loss temperatures of sucrose and ADP were roughly 195 and 150 °C, respectively [24]. Observation of such a dramatically lowered temperature may possibly be due to the catalysis by ADP towards thermal degradation or caramelization of the sucrose [30]. From the DTG curves, it could be seen that the SADP adhesives exhibited a one-step thermal degradation at around 150–158 °C, another temperature that was decidedly lower than the decomposition temperatures of neat sucrose and ADP. This too indicates that some reactions occurring during the synthesis process allowed for a new material to be generated that had vastly different thermal behavior than the original precursors. Compared with previous research, the degradation temperature of a sucrose-ADP mixture (without synthesis process) was 128 °C [24], which was also lower than the synthesized SADP adhesives used in this work. This was possibly due to the increasing polymerization degree of synthesized SADP adhesives. In addition, it was found that when prolonging synthesis time, a slightly decreased degradation temperature was observed. This indicates that the curing temperature of synthesis SADP adhesives were influenced by synthesis time.

**Figure 6:**
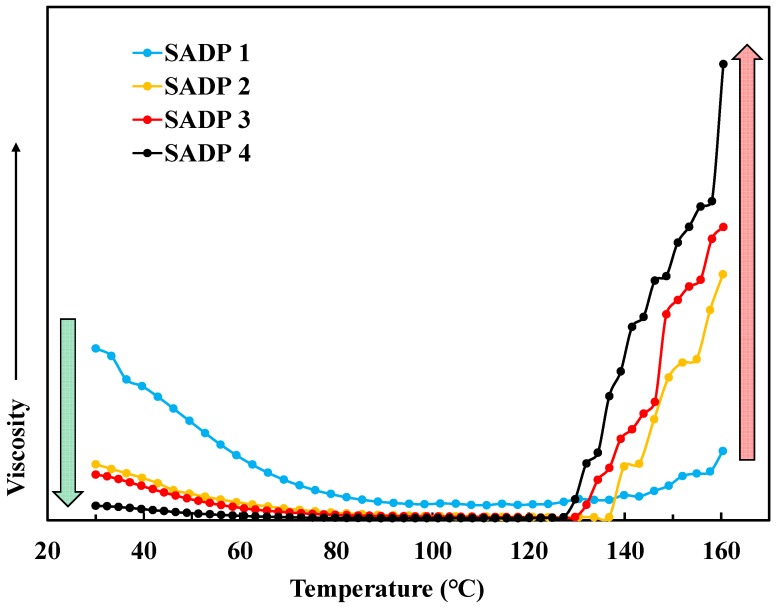
Viscosity-temperature characteristics of SADP adhesive.

To better clarify the intriguing TG analysis previously discussed, an effort was made to better understand the viscosity-temperature characteristics of the SADP adhesives. Results are shown in Figure 6. It is important to emphasize that due to the different measurement method, the viscosity results shown in Figure 6 are not accurate values. Instead, these results should be analyzed through the lens of understanding the trend of change for each adhesive′s viscosity over the tested temperature range.

First, it can be seen that when increasing the heating temperature up to 80 °C, the viscosity of each SADP adhesive predictably decreased until it became stable. However, at approximately 130 °C a rapid viscosity increase was observed. It is possible that the driving force for this instead was the onset of polymerization chemistry. Interestingly, only SADP 1 exhibited a moderate viscosity increase from approximately 135–160 °C, indicating that the curing process of SADP 1 was insufficient in this temperature range. Furthermore, initial viscosity values were found to be decreased when prolonging synthesis time. Judging from the similar trend observed in the TG analysis above (degradation temperature slightly decreasing with prolonged synthesis times), it seems that the extension of the synthesis time resulted in SADP adhesives that already contained some oligomeric or low molecular weight polymeric character. When looking only at the viscosities acquired at 160 °C, it was also found that these values increased with synthesis time. This too suggests that the rate of polymerization was positively correlated with adhesive synthesis time. 

All of the above results clarified that degradation reactions occurred in the SADP adhesive systems that drove up viscosity, indicating a polymeric substance formed during the heating treatment. Furthermore, according to the results of shear strength of the finished plywood, this polymeric substance contributed in some part to the favorable bond strength and water resistance of the wood products.

### 3.3. Synthesis Mechanism

#### 3.3.1. HPLC Analysis

The contents of the glucose and 5-HMF present within the SADP adhesives were measured by HPLC, and the results are shown in Table 4. First, it can be seen that glucose content decreases with prolonged synthesis times. As expected, accompanying this decrease is an elevation in 5-HMF concentration. This observation is best explained by the occurrence of sucrose hydrolysis during heating, which then allows for the liberated hexose monosaccharides to dehydrate hydrolyzed products (glucose and fructose) to 5-HMF. It is important to note that the above reaction is clearly one of the reactions taking place during the synthesis process, as judged by the appearance of the SADP adhesives (Figure 2). The brown coloration that continues to build up in the pictured materials indicates that some browning polymers also formed in the adhesives [25,26,31].

#### 3.3.2. ATR FT-IR Analysis

**Figure 7:**
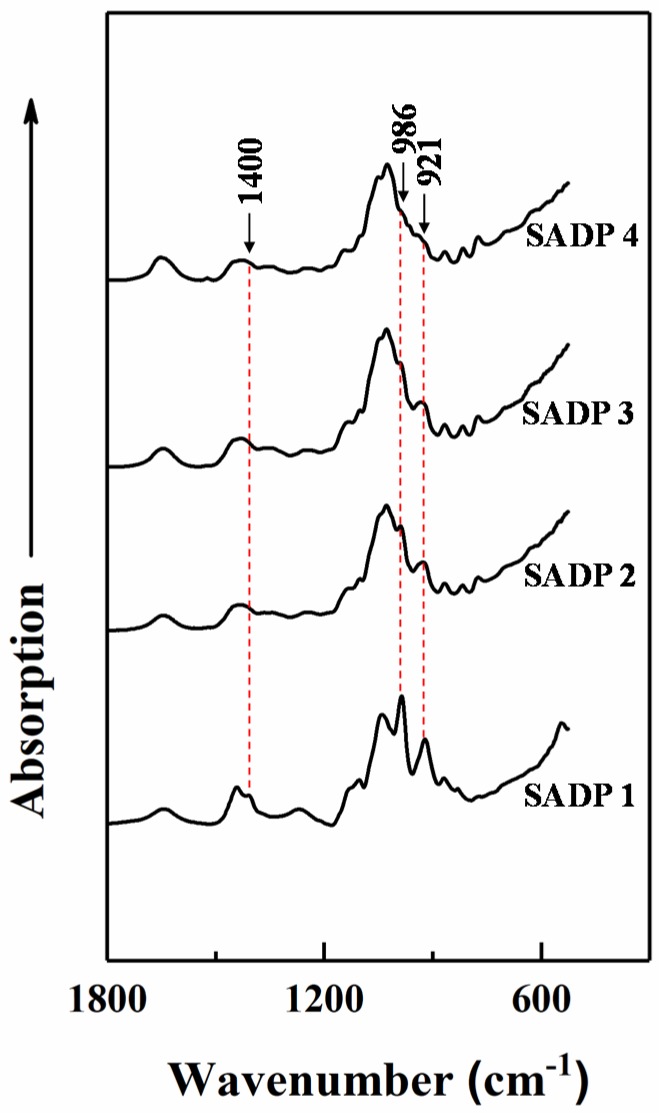
ATR FT-IR spectra of synthesized SADP adhesives after freezing dry.

To understand how the molecules in the adhesive systems transform over extended synthesis times, we chose to produce ATR FT-IR spectra of the synthesized SADP adhesives. Results are shown in Figure 7. It can primarily be observed that there were three noticeable peaks (1400, 986, and 921 cm^−1^) that either disappeared or decreased in intensity over synthesis time. The peak at 1400 cm^−1^ was attributed to the ammonium cation [32], which was only detected in the SADP 1, while it disappeared from each subsequently analyzed SADP adhesive. This indicates that the ammonium ion was consumed when the synthesis time was equal to or more than 2 h, and this was possible due to the hydrolysis of the ammonium ion [33]. Next, the peaks at around 986 and 920 cm^−1^ that were derived from the products of sucrose hydrolysis were asscribed to –OH groups [34] and pyranose rings [35], respectively. Both of these peaks decreased in signal intensity with prolonged synthesis times, possibly due to the dehydration reactions taking place during synthesis. 

As discussed, when qualitatively observing the synthesized SADP adhesives (Figure 2), some browning polymers formed during the synthesis process. In any system involving sucrose, it is known that caramelization, Maillard reactions, and the reducing sugars-ammonium reactions could each explain the formation of these brown solutes. Therefore, the characteristic products of the Maillard reaction and the reducing sugars-ammonium, such as amion compounds [36], Schiff bases [36], and deoxyglucosine [25], are also expected to be present within the synsized adhesives.

### 3.4. Curing Mechanism

#### 3.4.1. XPS Analysis 

**Figure 8:**
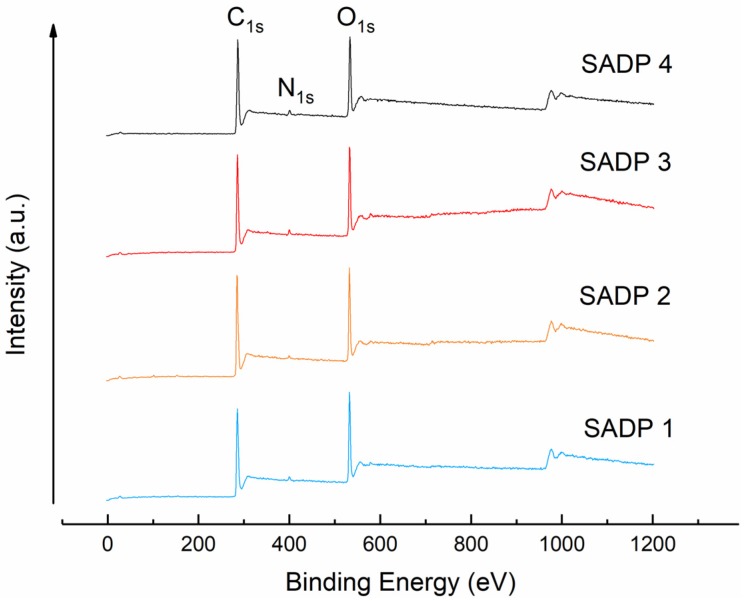
XPS spectra of synthesized SADP adhesives after freezing dry.

**Figure 9:**
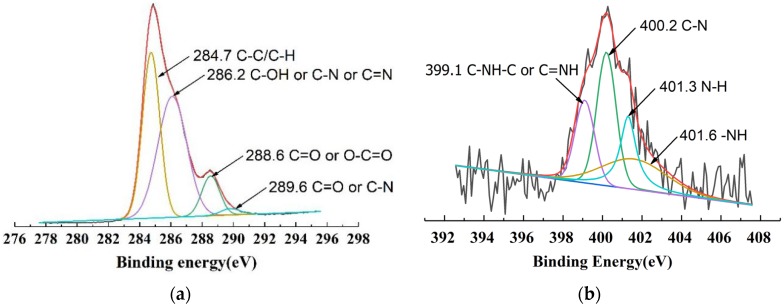
High-resolution XPS C 1s (**a**) and N 1s (**b**) spectra for the insoluble matter of SADP 2.

In our previous research, we recognized the existence of elemental nitrogen within the cured adhesive, which served as a significant clue towards developing a better understanding of the SADP adhesive’s curing mechanism within particleboard [24]. To further investigate this mechanism, in this work we chose to employ X-ray photoelectron spectroscopy (XPS) to further clarify the role that ammonium plays, and to learn how elemental nitrogen comes to be included within the finished product. Figure 8 shows the binding energy of the insoluble matter of each of the four tested adhesives. Specifically, the insoluble matter samples were obtained by first heating the liquid adhesive at 170 °C for 7 min, and next, by boiling the material in a water bath for 4 h. After the thermal treatments, the resultant mixture dried at 60 °C for 15 h, with the resultant solids serving as the substrate used for XPS analysis. From the results, it was observed that signals pertaining to C, N, and O all appeared across each tested specimen. In addition, the atomic mass content of C, N and O in insoluble matter derived from all the SADP adhesives were nearly the same. These values were approximately 74%, 2%, and 24%, respectively. It seems that the chemical elements composition of the insoluble matter was generally similar regardless of synthesis time. However, the spectra of C 1s and N 1s belonging to the insoluble matter from SADP 2 were further de-convoluted into four Gaussian peaks. These peaks represent the carbon-related and nitrogen-related chemical moieties, and from these peaks it is possible to develop a preliminary understanding of these chemical structures. Results from this analysis are shown in Figure 9. Regarding the de-convoluted C 1s peak (Figure 9a), binding energy (BE) at 284.7 eV is believed to be derived from both C–C and C–H bonds, two covalent linkages that clearly serve as the dominate bond within the insoluble cured polymer [37]. The signal at 286.2 eV reveals the existence of a C–OH, C–N, or C=N bond [38,39,40]. The peaks located at 288.6 and 289.6 eV were also considered to belong to carbonyl groups (C=O) or O–C=O [41,42], carboxylic groups (C=O), or C–N bonds [43]. The N 1s XPS of SADP 2 also could be separated by peak fitting into four spectral components, and the results are shown in Figure 9b. The BE signal located at 399.1 eV was attributed to C–NH–C or C=NH groups [44,45]. The peaks observed from 400.2 and 401.3 eV were common signals of C–N and N–H [46,47], respectively. The BE that appeared at 401.6 eV was possible due to –NH [48].

The dense yet informative results provided from XPS analysis again demonstrated the existence of fixed N within the cured SADP adhesives, and its mass could be approximated to be 2%. A preliminary chemical group analysis could detect the C=O, C–N, and C=N bonds, which are also commonly formed in the products of the Maillard reaction and sugars-ammonium reaction [25,36]. However, it is not possible to fully assess the diversity of chemical linkage in the curved adhesive by XPS analysis only; therefore, an additional FT-IR analysis was also carried out to further paint the picture of the actual structures and functionalities that comprise cured SADP adhesives.

#### 3.4.2. ATR-FTIR Analysis 

**Figure 10:**
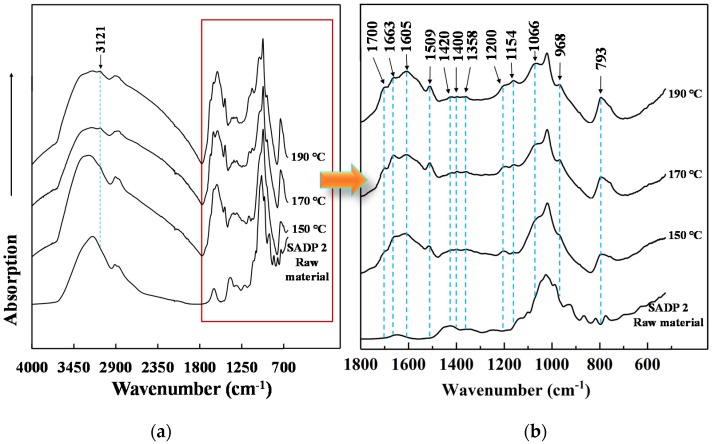
ATR FT-IR spectra of (**a**) insoluble matter derived from SADP 2 heated at different temperatures. (**b**) Fractionated gain from 1800–500cm^−1^.

In effort to further clarify the chemical structural changes experienced by our SADP adhesives over the course of the curing process, we recorded ATR FT-IR spectra to serve as a complimentary information source alongside the previously discussed XPS findings. Figure 10a shows the adsorption bands exhibited by the uncured SADP 2 and the insoluble masses obtained from SADP 2. In addition, the informationally dense region of 1800–500 cm^−1^ has been enlarged and can be found in Figure 10b. Compared with the uncured SADP adhesive, 13 new peaks were generated in the cured adhesives. The peaks located at around 3121, 1509, and 793 cm^−1^ were possibly due to new C–H and C=C stretching vibrations, as well as the CH=CH bonds present within resultant furan rings [24,49,50]. In addition, these absorption bands increased in resolution when increasing the heating temperature, indicating the curing process along with the polymerization of the furan compounds. Also, a new peak at around 1700 cm^−1^ could be found in the cured material, something that was attributed to the carbonyl functionalities [51]. Meanwhile, the absorption band at 1605 cm^−1^ was attributed to aromatic C–C strength, and this band is ubiquitous in the spectra of coals, chars, and several other heat-treated carbonaceous materials [52]. The peaks at 1663 and 1154 cm^−1^ were assigned to a C=N group of imine [53] and C–N bonds [54]. In addition, the presence of a feature between 1358 and 1421 cm^−1^ strongly resembled those of C–N=C in imine (Schiff base) [54]. These groups confirmed the formation of Schiff bases during the curing process, and the imine linkage seems established by the polymerization between the Schiff base and the aldehydes compounds derived from the dehydration of sucrose. Moreover, the peaks located at 1200, 1066, and 968 cm^−1^ were considered as C–O–C stretching, C–O bending, and C–O symmetric stretching vibrations [55,56], respectively. These groups indicated that the dimethylene ether bridges seemed to be another significant linkage in the cured adhesive.

#### 3.4.3. Consideration of the Synthesis and Curing Mechanisms Involved in SADP Adhesive 

**Figure 11:**
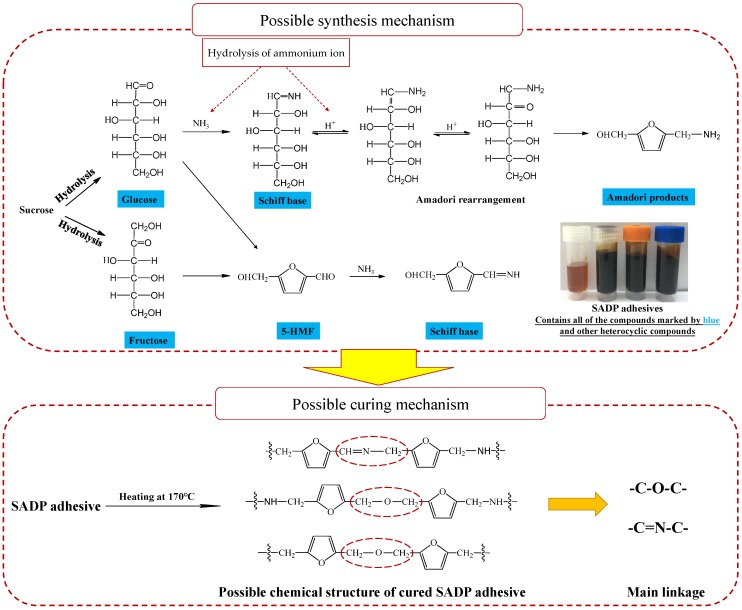
Possible synthesis and curing mechanism for SADP adhesive.

Based on the previous chemical analysis of both uncured and cured SADP adhesives, we have attempted to provide an overview of all the possible reactions that could take place during the synthesis and curing process, and the possible synthesis and curing mechanisms are shown in Figure 11. During the adhesive synthesis process, it appears clear that ammonium catalyzed the sucrose hydrolysis to glucose, and fructose initiated the formation where a part of the liberated hexoses were next converted to 5-HMF. Meanwhile, the heating treatment facilitated the hydrolysis of an ammonium ion to ammonium hydroxide, and possibly reacted with 5-HMF and the reducing monosaccharide to form heterocyclic compounds and a Schiff base by Amadori rearrangement. The synthesized SADP adhesives contain monosaccharide, 5-HMF, Schiff bases, and countless other presently undescribed reaction products. From this complex mixture, it appears that during the curing process, a series of dehydration condensation of the furan compounds and Maillard reactions also possibly take place to form a polymeric material, which is mainly composed of polyfuran and linked by C–O–C and C=N–C linkage. This final polymerized material is then capable of forming network adhesives upon woody substrates, resulting in the bio-based adhesives desired by both producers and consumers of wood products worldwide.

## 4. Conclusions

In this study, a novel, high solid content, eco-friendly wood adhesive was synthesized based exclusively on sucrose and ammonium dihydrogen phosphate (ADP). The intent behind the adhesive development was to use this as a bio-based alternative for implantation into various structural wood products. The effects of synthesis time and hot pressing temperature on the bonding performance were investigated. When the plywood was bonded with 2 h synthesized SADP adhesive, with hot pressing temperature 170 °C, hot pressing time for 7 min, and a spread rate at 140 g/m^2^ conditions, the wet shear strength was satisfied for the GB/T 9846-2015 standard. The curing behavior of SADP adhesives indicated that the mass loss temperature and viscosity were positively correlated with synthesis time. The synthesis mechanism showed 5-HMF existed within the uncured adhesives, and its amount increased over extended synthesis times. Other reactions, such as reducing sugar-ammonium reaction, hydrolysis of ammonium ion, Amadori rearrangement, caramelization, and Maillard reaction, also took place based on the observation of a brown coloration to the synthesized adhesives. XPS showed nitrogen participated in the curing reaction, and dimethylene ether bridges (C–O–C) and imine (N–C=N) were observed by IR spectrum. The main reactions during the curing process were considered as the dehydration condensation of furan compounds and Maillard reaction. 

This research is an initial study on this novel adhesion system. We provided the possibility to synthesize a high solid content wood adhesive by renewable materials for wood composites, which is expected to satisfy the necessity of the wood industry. However, some improvement methods such as reducing the hot pressing temperature, hot pressing time, and the effects of other synthesis and hot pressing conditions would be presented in our further research. 

## Figures and Tables

**Table 1:** Detailed information of SADP adhesives.

Adhesives	Proportion (Sucrose/ADP)	Synthesis Time (h)	Synthesis Temperature (°C)	Solid Content (%)	Viscosity (mPa·s)	pH
SADP 1	85/15	1	90	83	1266	3.48
SADP 2		2			788	2.95
SADP 3		3			621	2.35
SADP 4		4			476	2.01

**Table 2:** Manufacture conditions of the plywood.

Adhesives	Hot Pressing Temperature (°C)	Hot Pressing Time (min)	Spread Rate (g/m^2^)
	150		
SADP 1	170		
	190		
	150		
SADP 2	170		
	190		
	150	7	140
SADP 3	170		
	190		
	150		
SADP 4	170		
	190		

**Table 3:** Wood failure of the three-ply plywood bonded with SADP adhesives at different hot pressing temperatures.

Adhesives	Hot Pressing Temperature (°C)	Wood Failure Rate of Dry Condition (%)	Wood Failure Rate of Wet Condition (%)
SADP 1	150	0	0
	170	20	20
	190	80	55
SADP 2	150	0	0
	170	40	35
	190	85	55
SADP 3	150	5	0
	170	70	40
	190	95	90
SADP 4	150	10	0
	170	75	50
	190	100	100

**Table 4:** Wood failure of the three-ply plywood bonded with SADP adhesives at different hot pressing temperatures.

Adhesives	Glucose Content (g/L)	5-HMF Content (g/L)
SADP 1	714.67	1.00
SADP 2	616.23	9.32
SADP 3	503.41	12.96
SADP 4	457.82	49.84
